# Composite signet-ring cell/neuroendocrine carcinoma of the stomach with a metastatic neuroendocrine carcinoma component: a better prognosis entity

**DOI:** 10.1186/1746-1596-2-43

**Published:** 2007-11-07

**Authors:** Summer L Nugent, Steven C Cunningham, Borislav A Alexiev, Emily Bellavance, John C Papadimitriou, Nader Hanna

**Affiliations:** 1 University of Maryland Medical Center, Department of Pathology, NBW43, 22 S Greene Street, Baltimore, MD 21201, USA; 2 University of Maryland Medical Center, Department of Surgical Oncology, 419 West Redwood Street, Baltimore, MD 21201, USA

## Abstract

**Background:**

Mixed (composite) exocrine-neuroendocrine cell carcinomas are defined as an intimate admixture of neoplastic glandular exocrine and neuroendocrine cell types. Although gastric adenocarcinoma containing a small number of neuroendocrine cells is a relatively frequent occurrence, gastric neoplasms containing equal proportions of both cell types are rare.

**Case Presentation:**

We present a case of composite exocrine (signet-ring cell)-neuroendocrine cell carcinoma, in which the neoplastic signet-ring cell exocrine and neuroendocrine constituents occurred in fairly equivalent amounts, whereas only the neuroendocrine carcinoma portion of the tumor represented the metastatic component. Light microscopy, immunohistochemical and electron microscopic findings are described, and the literature is reviewed.

**Conclusion:**

This study confirms the ability of pluripotent precursor cells to differentiate into either adenocarcinoma or neuroendocrine tumor and, justifying the designation of composite exocrine-neuroendocrine cell carcinoma as the appropriate classification for this tumor. The protracted clinical course further supports the notion that composite signet-ring cell/neuroendocrine carcinoma tumors behave relatively less aggressively than the pure forms of the former cell type.

## Background

Adenocarcinoma and neuroendocrine carcinomas (NEC) are each well known to occur in the background of atrophic gastritis, but the concurrence of both adenocarcinoma and NEC (malignant carcinoid) together in the gastrointestinal tract (GI) is extremely rare [1,2]. Over the past several years, classification of these tumors has been a subject of debate. As described by Lewin and Appelman [3], mixed neuroendocrine and conventional carcinomas of the stomach should be classified in one of five distinct groups. These include: carcinomas with interspersed neuroendocrine cells, composite glandular-endocrine carcinomas, collision tumors, amphicrine tumors, and combinations of the above [1,3]. More recently, Fujiyoshi et al [4] reclassified the mixed endocrine and nonendocrine epithelial tumors by subdivision into six categories: 1) neuroendocrine cells interspersed within carcinomas; 2) carcinoids (neuroendocrine tumors/NET) with interspersed nonendocrine cells; 3) composite glandular-neuroendocrine cell carcinomas containing both areas of carcinoid and conventional carcinoma; 4) collision tumors in which neuroendocrine tumors and conventional carcinomas are closely juxtaposed but not admixed; 5) amphicrine tumors predominantly composed of cells exhibiting concurrent neuroendocrine and nonendocrine differentiation; and 6) combinations of the previous types. A variety of terms have been utilized in the designation of tumors along this continuum based on the predominant form of differentiation [5]. The occurrence of a composite tumor in the setting of atrophic gastritis has seldom been reported in the literature [6]. In adenocarcinomas of the gastrointestinal tract, the presence of both neuroendocrine and nonendocrine cells is a well-recognized finding [7]. However, mixed exocrine-neuroendocrine cell carcinomas are exceptionally rare. To our knowledge, only 15 cases of gastric mixed exocrine-neuroendocrine cell carcinomas have been reported in the literature (Table 1). Composite, or mixed glandular-neuroendocrine cell carcinomas, are defined as an intricate admixture of both elements which are present in equal proportions, unlike amphicrine tumors which demonstrate dual differentiation (exocrine and neuroendocrine) within the same cell. Moreover, collision type neoplasms occur as exocrine and neuroendocrine tumors that arise adjacent to one another and do not originate from the same cell type [8]. We review this literature, and present a case of a composite glandular-neuroendocrine carcinoma of the stomach emphasizing the light microscopy, immunohistochemical and electron microscopic characteristics of the tumor as well as the clinicopathological correlations.

**Table 1:** Review of gastric composite tumor literature

**First Author (ref)**	**Year**	**Type**	**Ca type**	**Out-come**	**Size (cm)**	**LN (+ of total)**	**EM**	**Synp**	**Chr**	**E-cad**	**any CK**	**Ki-67**
Nagoaka [13]	1996	Cmp	NR	DOD	8	NR	NR	NR	A-; NE+	NR	NR	NR
Fujiyoshi [4]	2004	Cmp	sign	NED @ 9 y	NR	0 of 13	NR	A-; NE-	A+; NE+	A- NE+	A-; NE-	NR
Fujiyoshi [4]	2004	Cmp	sign	NED @ 8 y	10.5	0 of 9	NR	A-; NE-	A+; NE+	A- NE+	A-; NE-	NR
Adhikari [6]	2002	Cmp	int	NED @ 1 y	6	NR	NR	A+f; NE+	A+f; NE+	A+f; NE+	A+; NE-	NR
Caruso [7]	1989	Cmp	int	NR	1.5	NR	NR	NR	A+; NE+	NR	NR	NR
Yang [10]	1991	Cmp/Amp	int	NED @ 6 mo	3	18 of 19	NE/M same	NR	+	NR	NR	NR
Ulich [14]	1988	Cmp	int	NR	3.6	NR	NR	NR	A+f; NE+	NR	NR	NR
Wheeler [15]	1983	Cmp	int	DOD @ 3 mo	5	NR	NR	NR	NR	NR	NR	NR
Ali [16]	1984	Cmp	sign	DOD	22	0 of 5	NE/M same	NR	NR	NR	NR	NR
Brouland [17]	2001	Cmp/Amp	sign	NED @ 6 mo	NR	NR	NE/M same	NR	+	NR	NR	NR
Jain [18]	2005	Cmp	int	LTFU	1.5	NR	NR	A+f; NE+	A+f; NE+	NR	A+; NE-	NR
Jain [18]	2005	Cmp	int	NED @ 2 y	3.3	NR	NR	A+f; NE+	A+f; NE+	NR	A-; NE-	NR
Jain [18]	2005	Cmp	int	DUC @ 4 mo	NR	NR	NR	A+f; NE+	A+f; NE+	NR	NR	NR
Klappenbach [5]	1985	Cmp	NR	NR	NR	NR	NR	NR	NR	NR	NR	NR
Pasquinelli [19]	1993	Cmp/Amp	NR	DOD	3	NR	NE/M same	+	+	NR	NR	NR

Present case	2007	Cmp	sign	NED @ 6 mo	3.5	3 of 12	NE/M diff	A+; NE+	A+; NE+	A+; NE-	A+; NE-	A+; NE-

Abbreviations: +, positive immunolabeling; -, negative; A, adenocarcinoma; Amp, amphicrine; Ca, carcinoma; Chr, chromogranin A; CK, cytokeratin; Cmp, composite; DOD, dead of disease; DUC, dead of unrelated causes; E-cad, e-cadherin; EM, electron microscopy; f, focal; diff = different. intest = intestinal; LN, lymph nodes; LTFU, lost to follow up; M, mucous; NE, neuroendocrine; NED, no evidence of disease; NR, not reported; sign = signet; Synp, synaptophysin;

## Case presentation

A 64-year-old Caucasian woman with a history of a resected gastric carcinoid 38 years ago presented to the Department of Gastroenterology at the University of Maryland with symptoms of early satiety and a progressive, 6-months weight loss of 20 pounds. The patient had no other complaints. Her past medical history was significant for trigeminal neuralgia which had been surgically treated. She did not take any medications and denied a family history of cancer. On exam the patient was a thin, well-appearing woman without remarkable abnormal physical findings. Laboratory values revealed a mild anemia with a hemoglobin of 10 mg/dL. Her electrolytes, liver function tests, and coagulation panel were within the normal ranges.

The patient underwent upper endoscopy and endoscopic ultrasound (EUS), which revealed the following findings: an area of nodular mucosa in the distal esophagus at the gastroesophageal junction, an erythematous patch in the stomach, a 2-cm gastric polyp, which was then resected, and an extragastric mass, consistent with a group of matted lymph nodes, located between the left lobe of the liver and the anterior wall of the stomach. The pathology results of the biopsied sites showed that the gastric polyp and nodular mucosa represented tubular adenomas, the latter containing focal intramucosal adenocarcinoma. The fine needle aspiration (FNA) of the gastric mass was consistent with metastatic NEC (malignant carcinoid), and the erythematous patch in the stomach at 40-cm showed invasive poorly differentiated/signet-ring cell carcinoma.

The patient underwent a staging computed tomography (CT) scan of the abdomen and pelvis that demonstrated two large enhancing masses between the left lateral lobe of the liver and the anterior wall of the stomach corresponding to the extragastric mass seen on endoscopic ultrasound. There was no evidence of distant metastases on CT scan.

The patient was then referred to the Department of Surgical Oncology, for evaluation for surgical resection. Intraoperatively, there was no evidence of distant metastases. A D2 total gastrectomy with omentectomy and regional lymph node dissection with a Roux-en-Y J-pouch esophagojejunostomy was performed. There were no palpable masses in the stomach. However, there were two palpable masses in the gastrohepatic ligament, consistent with metastatic lymph nodes. The spleen and distal pancreas were preserved.

Postoperative recovery was uneventful with the exception of one urinary tract infection. The patient was discharged to a rehabilitation facility on the fifteenth postoperative day.

### Materials

The use of paraffin blocks for this study meets Institutional Review Board and Health Insurance Portability and Accountability Act requirements, and has been approved by the Institutional Review Board at the University of Maryland Protocol Number: H-29227.

### Histology

The resected tissue was fixed in 10% buffered formalin and embedded in paraffin. The tissue was sectioned in 5 micron thick slices and stained with hematoxylin and eosin (H and E), mucicarmine and Periodic Acid-Schiff (PAS).

### Immunohistochemistry

Immunohistochemical staining was performed using Ventana Enhanced DAB Detection Kit and Biotin-StreptAvidin (B-SA) amplified methodology (Ventana, Tucson, AZ) and commercially available prediluted monoclonal antibodies against the following antigens: neuron specific enolase (NSE), synaptophysin, chromogranin, E-cadherin, pancytokeratin, CAM 5.2, and Ki-67 (all Ventana, Tucson, AZ).

### Electron microscopy

Fragments of formalin fixed gastric mucosa were also processed for evaluation by electron microscopy. Representative tissue samples (1-mm cubes) were fixed in 4F1G for 4 hours, postfixed in osmium tetroxide, dehydrated in graded alcohols, and embedded in epoxy resin. The sections were stained with uranyl acetate and lead citrate and examined on a JEM 1200 transmission electron microscope.

### Pathologic findings: gross appearance

Macroscopically, the gastrectomy specimen contained a tan-white plaque-like mass measuring 3.5 × 3.4 × 0.5 cm located in the body of the stomach (lesser curvature), approximately 10.5 cm from the proximal margin and approximately 15.2 cm from the distal margin, which appeared to invade both the mucosa and the submucosa. The uninvolved gastric mucosa was tan with pinpoint hemorrhages, with absence of folds. Also present were multiple, well-circumscribed, firm, yellow nodules (ranging in size 0.4–0.7 cm in greatest diameter) scattered throughout the mucosa and submucosa of the cardia and body of the stomach. There were also two larger extragastric nodules (5.5 × 2.7 × 3.1 cm and 7.2 × 3.8 × 2.9 cm respectively) consistent with matted lymph nodes that were tan, firm, and well-circumscribed and grossly abutting the wall of the lesser curvature. These two nodules were located 9.1 cm from the proximal margin and 13.2 cm from the distal margin respectively. The cut surfaces of the extragastric nodules were firm, pale tan to pink, and hemorrhagic. The attached adipose tissue contained multiple lymph nodes, ranging from less than 0.1 × <0.1 cm × <0.1 cm to 1.1 × 0.4 × 0.2 cm. Ten resected lymph nodes, in addition to the two large collections of matted lymph nodes, were submitted.

## Results

### Light microscopic appearance

**Figure 1:**
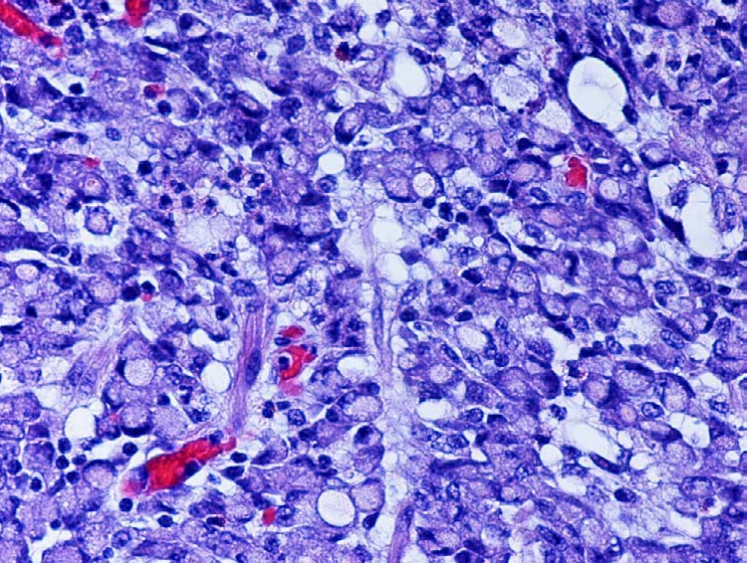
Representative section of the tumor showing the signet-ring cell adenocarcinoma component. H and E. Original magnification, × 400.

**Figure 2:**
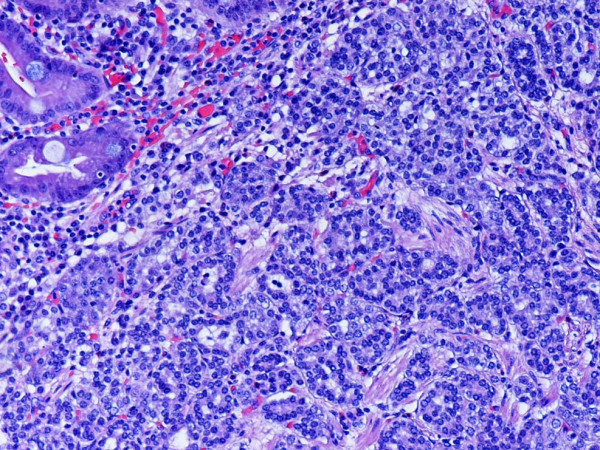
Representative section of the tumor showing the neuroendocrine carcinoma component. Intestinal metaplasia is noted in the upper left of the photo. H and E. Original magnification, × 200.

Microscopically, routine hematoxylin-eosin-stained sections from the tumor showed two distinct neoplastic phenotypes. The first type was composed of gastric-type epithelial cells with a high nuclear to cytoplasm ratio that diffusely permeated the mucosa and wall in an "infiltrative" growth pattern. The cytomorphology of these cells was characterized by large cytoplasmic mucin vacuoles and hyperchromatic eccentrically displaced nuclei. This component of the tumor was diagnosed as signet-ring cell adenocarcinoma (Figure 1). The second type was composed of smaller neoplastic cells located within the mucosa and submucosa that formed discrete islands, trabeculae, strands, glands, or solid sheets. These tumor cells were small and uniform with scant, eosinophilic granular cytoplasm and a centrally located round nucleus with finely dispersed ("salt and pepper") chromatin. This second component was diagnosed as NEC (malignant carcinoid) (Figure 2). Single neuroendocrine cells were noted to contain cytoplasm displaying a vacuolated appearance. Both components of the tumor invaded the subserosal layer. Lymphatic invasion was present but neither venous nor perineural invasion was noted. Metastatic NEC (malignant carcinoid) was found in three of the twelve lymph nodes. No evidence of metastatic signet-ring cell adenocarcinoma was found. Diffuse neuroendocrine cell hyperplasia and chronic gastritis with associated intestinal metaplasia were additional pathologic findings noted in the surrounding nonneoplastic mucosa of the stomach. Proximal and distal margins were negative for tumor.

### Special stains

**Figure 3:**
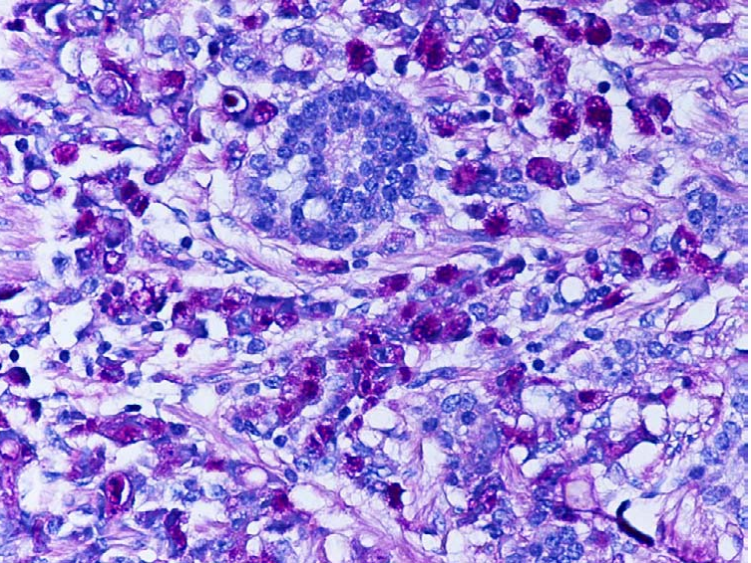
Representative section of the tumor showing PAS positive signet-ring carcinoma cells and PAS negative neuroendocrine tumor cells. PAS. Original magnification, × 400.

The results of these studies demonstrated that signet-ring adenocarcinoma cells were, to varying degrees, positive for mucopolysaccharides with mucicarmine and PAS stains, while the NEC component, including the neuroendocrine cells containing vacuolated cytoplasm, were completely negative (Figure 3).

### Immunohistochemistry

**Figure 4:**
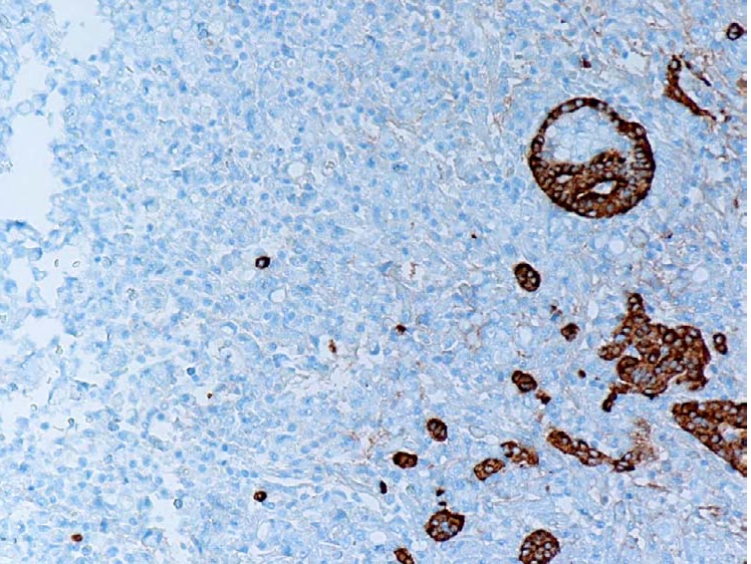
Representative section of the tumor showing synaptophysin positive neuroendocrine cells and synaptophysin negative signet-ring carcinoma cells. Anti-synaptophysin. Original magnification, × 100.

**Figure 5:**
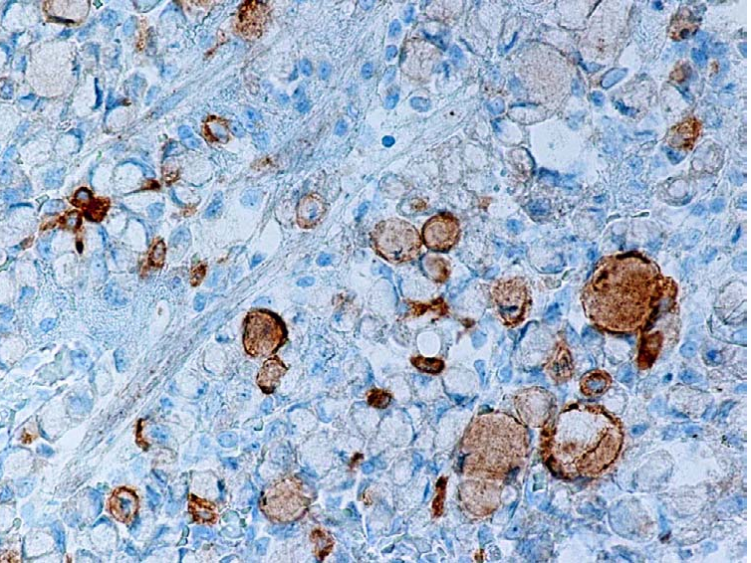
Representative section of the tumor showing single synaptophysin positive signet-ring carcinoma cells. Anti-synaptophysin. Original magnification, × 400.

**Figure 6:**
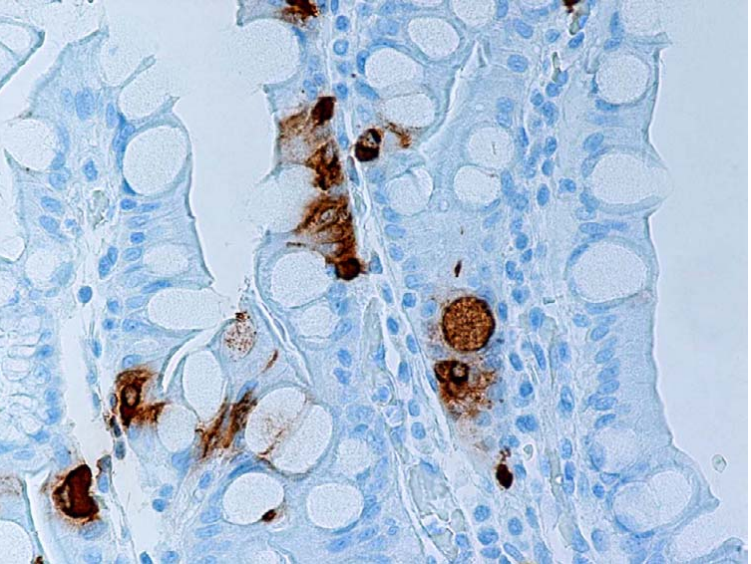
Representative section of gastric mucosa with intestinal metaplasia. Neuroendocrine cells and single goblet cells stain positive for synaptophysin. Anti-synaptophysin. Original magnification, × 400.

**Figure 7:**
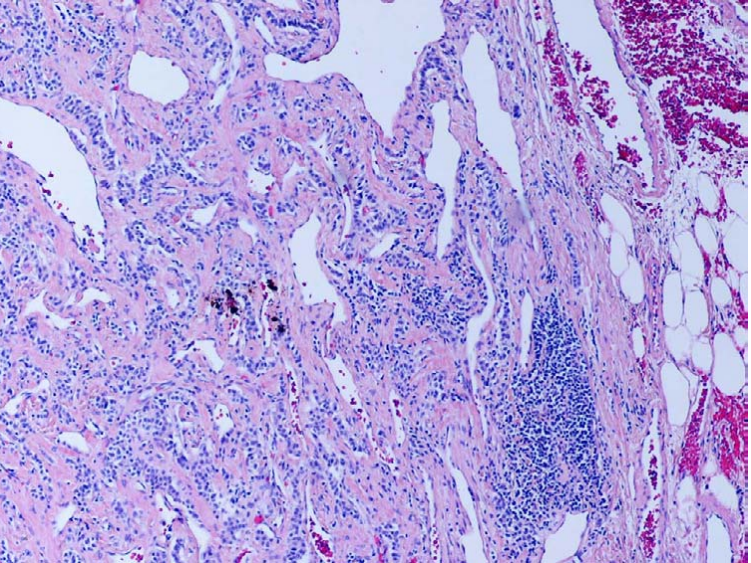
Representative section of neuroendocrine carcinoma component lymph node metastasis. H and E. Original magnification, × 100.

**Figure 8:**
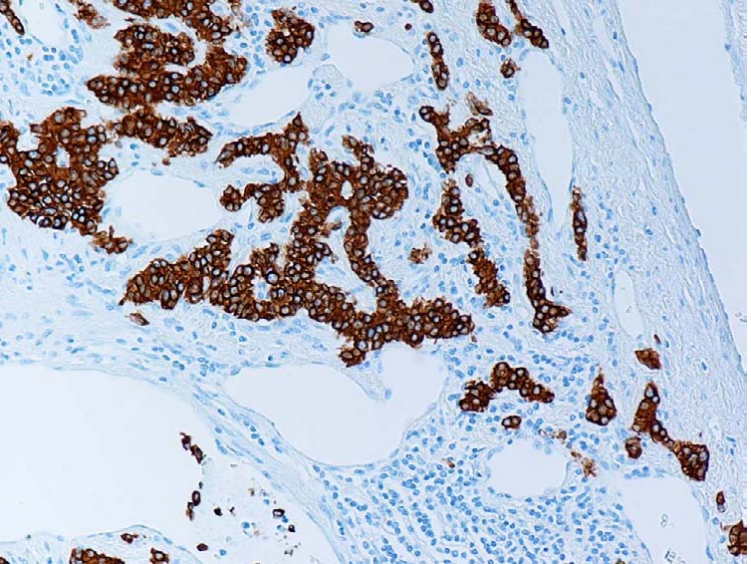
Representative section of lymph node metastasis. Note strong expression of synaptophysin in tumor cells. Anti-synaptophysin. Original magnification, × 100.

Immunohistochemistry studies demonstrated that the NET was strongly positive for chromogranin-A, synaptophysin and NSE, while the majority of signet-ring cells were negative (Figure 4). Pancytokeratin, E-cadherin, and CAM 5.2 immunolabeling was present in both the signet-cell adenocarcinoma and the neuroendocrine tumor components. Occasional scattered signet-ring carcinoma cells and goblet cells in the nonneoplastic metaplastic gastric mucosa stained positive for synaptophysin and chromogranin A (Figures 5 and 6). Ki-67 immunolabeling revealed a significant proliferation index (>70%) in the signet-ring cell carcinoma component of the tumor. In contrast, only rare neoplastic neuroendocrine cells expressed Ki-67 (<2%). The lymph node metastases stained diffusely and strongly positive for synaptophysin and chromogranin A (Figures 7 and 8), consistent with the light microscopic classification of these as neuroendocrine carcinoma exclusively.

### Electron microscopy

**Figure 9:**
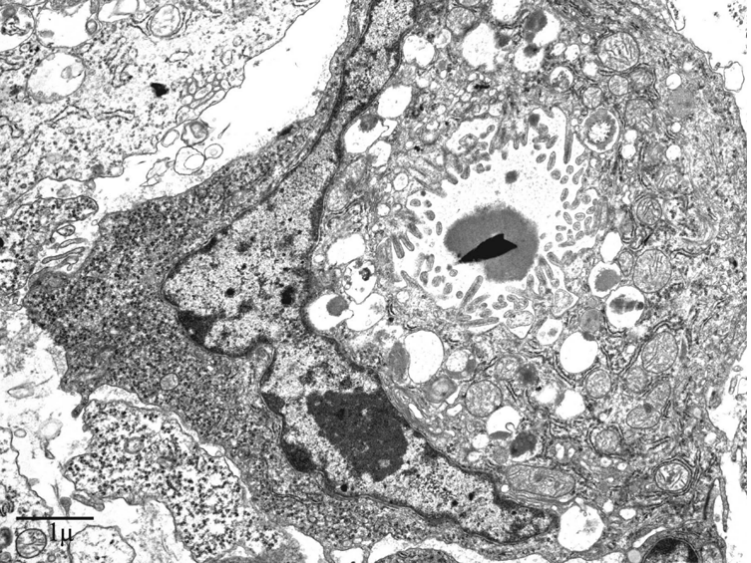
Representative section of signet-ring carcinoma cell. Note the presence of intracellular lumen lined with microvilli. EM. Original magnification, × 5000.

Electron microscopically, the signet-ring carcinoma cells possessed a hyperchromatic nucleus peripherally displaced and compressed by moderately electron-dense mucous granules. The cytoplasmic organelles were decreased and could be seen at the periphery of the cell or between mucous granules. Intracellular lumens lined by microvilli were frequently observed (Figure 9).

**Figure 10:**
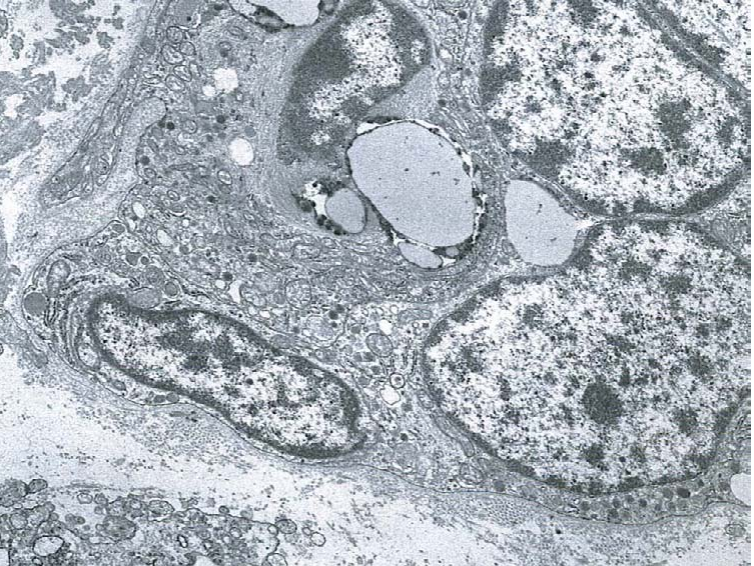
Representative section of neuroendocrine carcinoma cells. Note the presence of electron-dense endocrine granules tightly surrounded by a membrane and the presence of focal lipid deposits. EM. Original magnification, × 8000.

The nuclei of the neuroendocrine cells were oval or round. A narrow-to-chunky border of chromatin along the nuclear periphery was seen to surround small nucleoli. Endoplasmic reticulum, Golgi complex, free ribosomes, mitochondria and lysosomes occurred quite regularly. The neuroendocrine granules were round or oval, and consisted of homogeneous, electron-dense areas tightly surrounded by a membrane. Also present were single neuroendocrine cells containing lipid deposits, but no mucin, which corresponded to the clear cell signet-ring like vacuolated appearance of the tumor cell cytoplasm previously evident in some NEC cells in the H and E stained section (Figure 10). Although immunohistochemical stains demonstrated a minute number of cells displaying amphicrine features, i.e. cells that had both neuroendocrine and mucous granules, ultrastructural examination showed no evidence of cells containing both exo- and neuroendocrine features within the same cell.

## Conclusion

In the present study it was determined by light microscopy that the neoplastic cell population contained equal amounts of both tumor components, displaying on the one hand a typical signet-ring cell adenocarcinoma pattern admixed with clusters of small cells consistent with neuroendocrine differentiation on the other. The signet-ring cell carcinoma component showed positivity for pancytokeratin and CAM 5.2, as well as mucopolysaccharides but was negative for endocrine markers in the majority of neoplastic cells. The NEC portion of the neoplasm was positive for synaptophysin, chromogranin, NSE, pancytokeratin, and CAM 5.2. Only scattered rare single signet-ring cell carcinoma cells demonstrated amphicrine features (i.e. expression of both neuroendocrine immunohistochemical markers as well as positive mucicarmine and PAS staining), this being inconsistent with the diagnosis of a true amphicrine tumor. Furthermore, on ultrastructural examination, concurrence of mucous and endocrine granules within the same cell was not observed, whereas the presence of pure exocrine features e.g. intracellular lumina, was observed in the majority of the signet-ring cells with only a minority of them reaching the signet-ring cell morphology through lipid accumulation within an otherwise typical neuroendocrine cell. We thus concluded that the neoplasm contained two distinct cell types and therefore represents a composite signet-ring cell-neuroendocrine carcinoma.

The histiogenesis of these composite tumors remains unclear. It has recently been proposed that signet-ring cell carcinomas originate from the gradual dedifferentiation from enterochromaffin-like (ECL) cells through signet-ring cells with endocrine immunoreactivity [9]. In addition, two other hypotheses have been proposed, the first suggesting coincidental neoplastic changes in two different cell types and the second proposing a single common precursor cell that undergoes a bidirectional neoplastic change [10]. In the current case, the latter hypothesis appears to be better suited in accounting for the intimate admixture of the different cell types and patterns in this tumor. An interesting finding, supporting the above mentioned hypothesis, was the appearance of amphicrine cells in the signet-ring cell carcinoma component as well as in the adjacent nonneoplastic gastric mucosa.

Four types of "pure" neuroendocrine tumors (NET) can be distinguished in the stomach. Type 1 is the most common, occurring in 70–80% of all cases. In most cases, type 1 NETs of the stomach are small (0.1–1 cm in diameter), multifocal tumors, affecting women more than men, and always occurring in the background of chronic atrophic gastritis. Type 3 (sporadic and solitary) is the second most common, whereas types 2 (occurring in association with multiple endocrine neoplasia type 1) and 4 (undifferentiated solid neuroendocrine carcinoma) are considered rare [11]. The clinical setting and morphologic features of the NEC component in our case resemble the previously described clinicopathologic findings in type I gastric NETs [11]. Due to the fact that the NET component in the current case was significantly larger than 2 cm and had metastasized to a group of matted lymph nodes located adjacent to the lesser curvature of the stomach, we can hypothesize that this tumor would behave similar to a well-differentiated neuroendocrine carcinoma based on the WHO classification [11]. This was a very unusual finding, given that the NEC (malignant carcinoid) component was metastatic without any participation of the signet-ring cell component, even if the latter is typically the more malignant tumor. Previous studies have shown that regional lymph-node metastases occurred in very rare cases of type 1 NET in which the tumors were larger than 2 cm and infiltrated the muscularis propria [11]. One of the latest studies on gastrointestinal NETs demonstrated that small, low-grade NETs with low proliferation index may behave in a highly aggressive fashion, i.e. with multiple lymph node and hematogeneous metastases [12].

The general biologic behavior of gastric mixed exocrine-neuroendocrine neoplasms is unknown. A recent study suggests that composite tumors have a better prognosis than common gastric adenocarcinomas [2]. Certainly the course in our case represents a much less aggressive pattern than a classical signet-ring cell carcinoma. As recently emphasized in cases of advanced signet-ring cell carcinoma with better prognosis than expected [2], the diagnosis of composite glandular/neuroendocrine tumor needs to be considered.

## Competing interests

The author(s) declare that they have no competing interests.

## Authors' contributions

SLN processed the specimen, evaluated the immunohistochemical stains, confirmed the diagnosis, designed the report and drafted the manuscript.

BAA evaluated the immunohistochemical stains, confirmed the diagnosis, designed the report and drafted the manuscript.

SCC provided surgical intervention, relevant information and review of the literature.

NH and EB provided surgical intervention and relevant information.

JCP provided consultation.

All authors read and approved the final manuscript.
